# Differential Lipid Accumulation on HepG2 Cells Triggered by Palmitic and Linoleic Fatty Acids Exposure

**DOI:** 10.3390/molecules28052367

**Published:** 2023-03-04

**Authors:** Francisca S. Teixeira, Lígia L. Pimentel, Susana S. M. P. Vidigal, João Azevedo-Silva, Manuela E. Pintado, Luís M. Rodríguez-Alcalá

**Affiliations:** CBQF—Centro de Biotecnologia e Química Fina—Laboratório Associado, Escola Superior de Biotecnologia, Universidade Católica Portuguesa, Rua Diogo Botelho 1327, 4169-005 Porto, Portugal

**Keywords:** steatosis, hepatocytes, fatty acids, triglycerides, Oil Red O, lipid accumulation

## Abstract

Lipid metabolism pathways such as *β*-oxidation, lipolysis and, lipogenesis, are mainly associated with normal liver function. However, steatosis is a growing pathology caused by the accumulation of lipids in hepatic cells due to increased lipogenesis, dysregulated lipid metabolism, and/or reduced lipolysis. Accordingly, this investigation hypothesizes a selective in vitro accumulation of palmitic and linoleic fatty acids on hepatocytes. After assessing the metabolic inhibition, apoptotic effect, and reactive oxygen species (ROS) generation by linoleic (LA) and palmitic (PA) fatty acids, HepG2 cells were exposed to different ratios of LA and PA to study the lipid accumulation using the lipophilic dye Oil Red O. Lipidomic studies were also carried out after lipid isolation. Results revealed that LA was highly accumulated and induced ROS production when compared to PA. Lipid profile modifications were observed after LA:PA 1:1 (*v*/*v*) exposure, which led to a four-fold increase in triglycerides (TGs) (mainly in linoleic acid-containing species), as well as a increase in cholesterol and polyunsaturated fatty acids (PUFA) content when compared to the control cells. The present work highlights the importance of balancing both PA and LA fatty acids concentrations in HepG2 cells to maintain normal levels of free fatty acids (FFAs), cholesterol, and TGs and to minimize some of the observed in vitro effects (i.e., apoptosis, ROS generation and lipid accumulation) caused by these fatty acids.

## 1. Introduction

In 2019, the worldwide incidence of non-alcoholic fatty liver disease (NAFLD) was one hundred and seventy million, corresponding to twice the number of cases registered in 1990 [1]. One in three adults and one in ten children in the United States have hepatic complications regarding NAFLD, which currently has no approved pharmacological treatment. Consequently, liver transplantation will continue to represent the most efficient procedure to treat this disorder [2,3]. The aforementioned condition (i.e., NAFLD) is associated with intrahepatic lipid accumulation (5% compared to healthy individuals). The accumulation of intracellular triglycerides via lipid droplet (LD) formation is correlated to increased susceptibility to oxidative and cytokine stress, as well as inflammatory and hepatic injury characterized by NAFLD complications commonly referred as non-alcoholic steatohepatitis (NASH) [4,5]. Usually, the pathology evolves from fatty liver to NASH, and the main consequences are hepatic fibrosis and cirrhosis [3,6]. The main risk factors of NASH are being overweight, obesity, alcohol consumption, type II diabetes, treatments involving glucocorticoids, and hepatitis C viral infection [6].

Higher free fatty acid (FFA) uptake, increased hepatic lipid accumulation, de novo lipogenesis, and endoplasmic reticulum (ER) stress are hallmarks of NAFLD development and progression [7,8,9]. Once released from adipocytes, FFA can result in hepatocyte dysfunction and dysregulated lipid metabolism [10]. Individually, FFAs exert different effects on physiological processes, including cellular membranes properties, lipolysis, inflammation, and endocrine signaling regulation. Consequently, modifications in such processes due to altered unsaturated/saturated fatty acids (FA) plasma levels can lead to the development of hepatocyte dysfunction [11,12,13].

Lipid droplets are cellular organelles that originate from the ER and consist of a core of neutral lipids (cholesterol esters and triglycerides) enveloped by phospholipids and proteins [3]. These storage vesicles have great influence on lipid metabolism and on the progression of NAFLD. The growth of LDs is dependent on triglyceride and enzymatic phospholipid synthesis, and its catabolism is related to TG hydrolysis (releasing fatty acids) via β-oxidation. Interestingly, lipase activity and additional TG hydrolysis is regulated by proteins complexed to LD, such as perilipins, which are correlated to peroxisome proliferator-activated receptor-α (PPAR-α) activity [14,15]. These receptors are ligand-activated nuclear transcription factors that bind and respond to different FFAs. Their binding to both saturated and unsaturated FFAs regulate, to a different extent, their uptake, oxidation, and inhibit of de novo fatty acid synthesis mainly in tissues that retain high fatty acid catabolism (e.g., liver, kidney, heart, and skeletal muscles) [11]. The increased oxidation of FAs triggers reactive oxygen species (ROS) formation, lipid peroxidation, DNA damage, mitochondrial dysfunction, and release of pro-inflammatory cytokines [3,6].

As the accumulation of lipids in hepatocytes is considered a pathologic hallmark, in vitro models of steatosis have been used to study the hepatocellular consequences of lipid accumulation in human hepatic cells. These models include hepatocyte cell lines and primary hepatocytes treated in culture with monounsaturated and saturated fatty acids in order to mimic and explore novel features of NAFLD [16].

Considering that Western diets are characterized by high intake of animal fats containing palmitic acid (PA) and seed oils with high linoleic acid (LA), this study aims to determine the effect and accumulation of PA and LA on hepatocytes [17,18,19]. Thus, LA and PA accumulation impact hepatocyte metabolic inhibition, apoptosis, ROS-generated species, and lipid profile, which were parameters assayed in this research work.

## 2. Results

### 2.1. Metabolic Inhibition of LA and PA on HepG2 Cells

**Figure 1:**
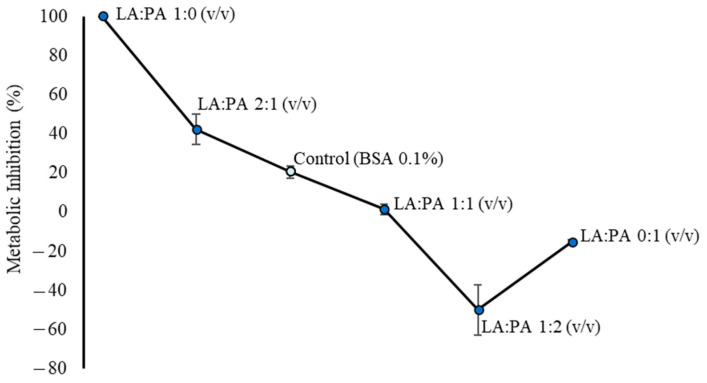
Metabolic inhibition (%) by PrestoBlue of 1 mM exposure to different ratios (*v*/*v*) of LA:PA on HepG2 cells. LA: linoleic acid, PA: palmitic acid.

To understand the interference of each fatty acid, saturated (palmitic acid—PA) and polyunsaturated (linoleic acid—LA), in the hepatocyte metabolic inhibition, the PrestoBlue assay was performed. Figure 1 represents the results obtained from the metabolic inhibition assay after the exposure of HepG2 cells to the mixture containing LA and PA at the concentration of 1 mM, varying the proportions (*v*/*v*) of these FFA.

As shown in Figure 1, a higher metabolic inhibition was observed for LA:PA 1:0 (*v*/*v*) ratio (approx. 100% vs. 20% in Control Cells). On the other hand, lower concentrations or the absence of LA in the 1 mM FFA mixture [LA:PA 1:1, 1:2 and 0:1 (*v*/*v*) ratios] were associated with lower metabolic inhibition values, namely, 1.54%, −50.01%, and −15.41%, respectively. These results indicate that LA induces higher metabolic inhibition than PA. However, such behavior may also be related to the proportion of dead, apoptotic, and live cells.

### 2.2. Flow Cytometric Analysis of LA and PA Effect on Apoptotic Stages

**Figure 2:**
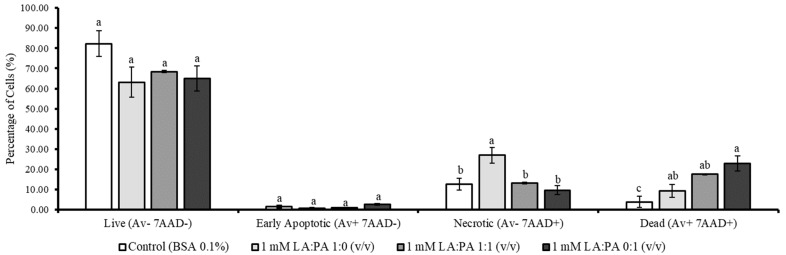
Live, early apoptotic, necrotic, and dead percentage of cells (%) after 1 mM exposure using different ratios (*v*/*v*) of LA:PA on HepG2 cells. BSA: bovine serum albumin, LA: linoleic acid, PA: palmitic acid; Annexin V (Av); 7-amino-actinomycin D (7AAD). Different letters for statistically significant differences within each group.

To evaluate if the metabolic inhibition results were in accordance with cell death, the apoptosis stages detection kit was used. The obtained percentage of live, early apoptotic, necrotic, and dead cells after the exposure to 1 mM LA:PA at different ratios (1:0; 1:1 and 0:1 *v*/*v*) are shown in Figure 2. Regarding the percentage of live cells, results suggested that no significant differences between exposure to FFA and the control group were found. Thus, the obtained values were 82.32 ± 6.33% for Control, 63.19 ± 7.39% for LA:PA at 1:0 (*v*/*v*), 68.46 ± 0.62% for 1:1 (*v*/*v*), and 64.98 ± 6.12% for 0:1 (*v*/*v*).

For early apoptosis, the percentages of live cells were: 1.40 ± 0.68% for Control, 0.73 ± 0.27% for LA:PA at 1:0 (*v*/*v*), 0.88 ± 0.19% for 1:1 (*v*/*v*), and 2.59 ± 0.30% for 0:1 (*v*/*v*) (*p* > 0.05). Moreover, LA (1 mM) exposure resulted in a higher level of necrotic cells (26.89 ± 3.92%) compared to the other assayed conditions (*p* < 0.05). Interestingly, the percentage of dead cells was higher when PA was in the mixture. The 0:1 (*v*/*v*) ratio, containing 1 mM of PA, significantly led to the highest percentage of cell death (22.85 ± 3.63%) when compared to control.

Linoleic and palmitic acids exert different effects on HepG2 cells, observed by both metabolic inhibition and apoptotic stages determination assays. Thus, from these two experiments, the observations suggest that LA may impair cell metabolism (Figure 1), while LA and PA might result in different cell death stages (Figure 2).

As these apoptotic events can be triggered by oxidative stress [10], and the assessment of intracellular reactive oxygen species (ROS) was evaluated for 1 mM of LA:PA at 1:0, 1:1, and 0:1 *v*/*v* ratios.

### 2.3. Assessment of Intracellular Reactive Oxygen Species (ROS)

**Figure 3:**
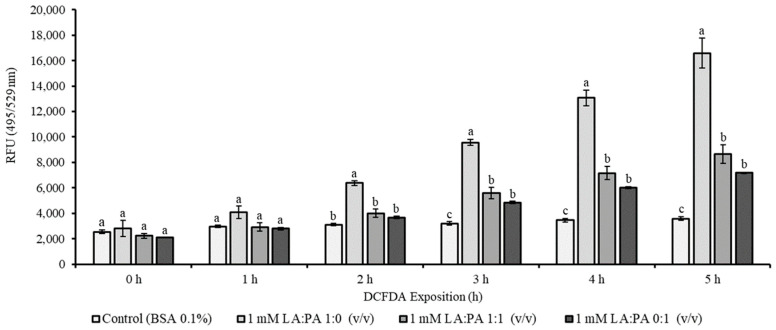
Relative fluorescence units (RFU) after DCFDA incubation during 5 h to predict intracellular HepG2 cells ROS generation after exposure to different ratios (*v*/*v*) of 1 mM LA:PA. LA: linoleic acid, PA: palmitic acid. Different letters for statistically significant differences within each group.

The assessment of intracellular reactive oxygen species (ROS) was evaluated to confirm if LA accumulation result in higher oxidative stress than PA in HepG2 cells. The results of relative fluorescence units (RFU) after exposure of hepatocytes to 2′,7′-dichlorofluorescein diacetate (DCFDA) for 5 h are depicted in Figure 3. The generated ROS were monitored after a 24 h exposure of hepatocytes to the different FFA ratios.

Depending on the nature of the FFA, different levels of ROS were observed. Cells exposed only to LA (LA:PA 1:0 *v*/*v*) showed an increase (*p* < 0.05) in ROS production after 2 h of DCFDA probe incubation when compared to the other ratios. Moreover, after 3 to 5 h of probe incubation, the presence of PA (LA:PA 1:1 and LA:PA 0:1 *v*/*v*) showed a higher (*p* < 0.05) RFU value when compared to the control. Hence, the mixture of 1 mM LA:PA 1:1 (*v*/*v*) resulted in a similar RFU value as the presence of LA:PA at 0:1 (*v*/*v*) (*p* < 0.05). After 5 h, superior ROS production and, therefore, higher RFU values, were achieved (*p* < 0.05) with the presence of LA (16589 RFU value), which was approximately the double of the RFU value obtained for LA:PA 1:1 and LA:PA 0:1 (*v*/*v)*, 8653 and 7180 RFU respectively. The reported results are in accordance with the hypothesis that, due to the presence of double bonds, LA is more prone to oxidation than PA on HepG2 cells.

### 2.4. Lipophilic Dye Oil Red O (ORO)

The parameters previously evaluated (e.g., metabolic inhibition, apoptotic stage determination, and ROS assessment) were of great relevance to discriminate some cellular effects of LA, PA, as well as the mixture of both FFA at equal concentrations. Besides these findings, and because PA is mostly associated with higher risk of cardiovascular diseases and as LA is an essential fatty acid [20,21], their accumulation on HepG2 cells was also studied.

**Figure 4:**
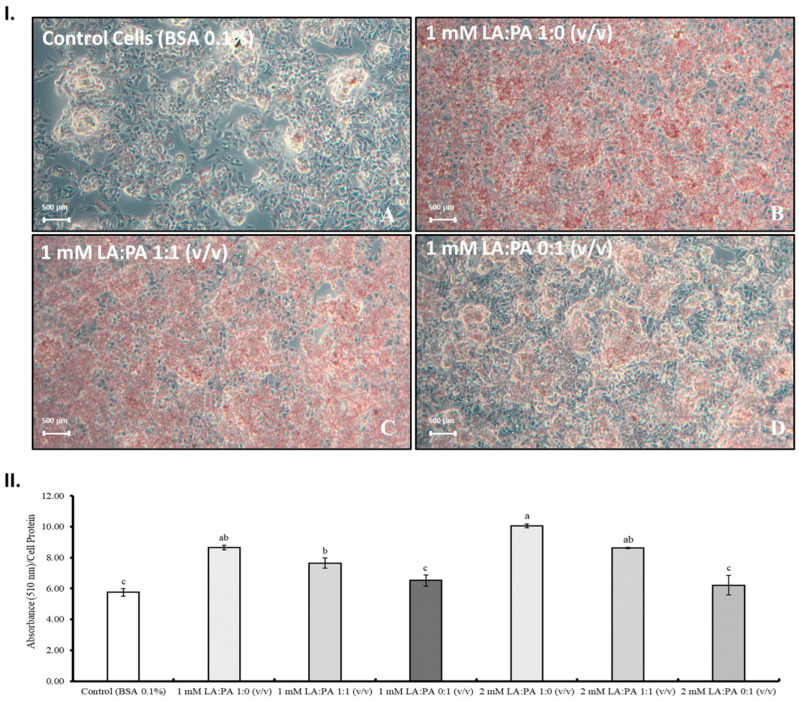
(**I**) Microscopic lipid accumulation visualization on HepG2 cells after FFA exposure, cell fixing, and staining; **A**—control cells; **B**—LA:PA 1:0 (*v*/*v*); **C**—LA:PA 1:1 (*v*/*v*) and **D**—LA:PA 0:1 (*v*/*v*). (**II**) Absorbance (510 nm)/cell protein) of HepG2 cells after 1 and 2 mM FFA exposure. LA: linoleic acid, PA: palmitic acid. Different letters for statistically significant differences.

Changes in cellular metabolism and lipid accumulation are increased by NAFLD and in vitro studies using ORO lipophilic dyes are usually used to predict lipid presence [5]. Hence, the visualization of HepG2 cells after FFA exposure and ORO staining can be seen in Figure 4I. In Figure 4II are expressed the ORO semi-quantification values after 1 and 2 mM FFA exposure using different ratios to confirm a possible dose-dependent effect. Results revealed some differences in lipid accumulation since, compared to control, higher LA concentrations led to higher lipid staining [Figure 4I(B) and Figure 4I(C)]. In Figure 4II, it is possible to observe that the normalized absorbance values of the control and LA:PA 0:1 (*v*/*v*) at 1 and 2 mM are not statistically different (*p* > 0.05), which indicates a low accumulation of PA independently of the assayed concentration (1 and 2 mM).

Based on the previously discussed results, the LA:PA 1:1 (*v*/*v*) ratio was selected to study the lipid profile of HepG2 cells, as this ratio increased lipid accumulation, as well as reactive oxygen species without metabolic inhibition impairment.

### 2.5. Total Fatty Acids Profile

The fatty acids (FA) content (mg FA/g cell pellet) obtained by GC with flame-ionization detector (GC-FID) is shown in Table 1.

The analysis of the total FA (free and esterified) profile, observed in the cell pellet after exposure to LA:PA 1:1 (*v*/*v*) indicates, in general, a significant accumulation in terms of total FAs (106.78 ± 0.11 vs. 256.66 ± 0.54 mg FA/g cell pellet on unexposed and exposed, respectively). Moreover, the FA profile showed that the LA was at lower concentrations (*p* < 0.01) in the control when compared to the exposed cells (1.12 ± 0.01 and 116.75 ± 0.26 mg FA/g cell pellet, respectively). Overall, PA and LA were the main fatty acids present but, after exposure, the PA concentration (60.77 ± 0.12 mg FA/g cell pellet) was approximately half of the LA concentration value (116.75 ± 0.26 mg FA/g cell pellet; *p* < 0.01).

Additionally, stearic (C18), cis-11,14-eicosadienoic (C20:2c11c14), cis-8,11,14-eicosatrienoic (C20:3c8c11c14), and arachidonic acid (C20:4c5c8c11c14) were other detected FAs with significantly increased concentrations when cells were cultured in the presence of the FFA mixture.

The results confirm that LA and PA are not equally accumulated and correlate with the previous observations of this research work concerning ORO staining results (Figure 4).

### 2.6. Free Fatty Acids, Cholesterol and Triglycerides Analysis by Gas Chromatography—Mass Spectrometry (GC-MS)

The performed FA analysis (Section 2.5.) allowed to quantify total FA independently if they were in the free form (i.e., FFA) or esterified (i.e., TG). Although LA and PA were exposed to the same concentration [LA:PA 1:1 (*v*/*v*)], data suggest that LA was highly accumulated. Thus, the following GC-MS analyses were performed to understand if this accumulation was as FFA or TG.

**Figure 5:**
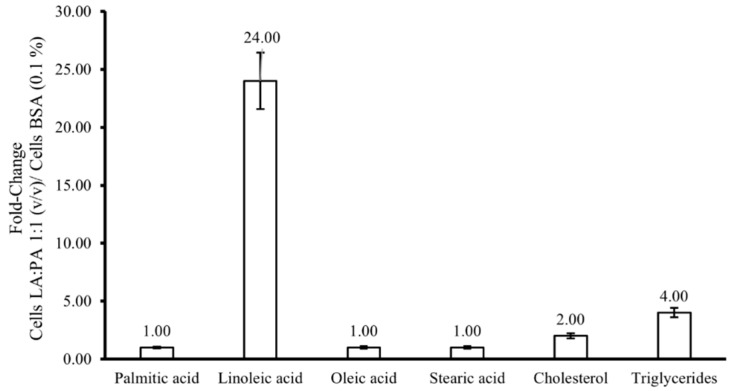
Fold change variation of the free palmitic, linoleic, oleic and stearic acids, cholesterol, and triglyceride content by GC-MS analysis after FFA exposure [LA:PA 1:1 (*v*/*v*)/control cells with BSA 0.1%]. FFA: free fatty acids, LA: linoleic acid, PA: palmitic acid.

The fold-change variation [LA:PA 1:1 (*v*/*v*)/control cells] of the free PA, LA, oleic and stearic acids, as well as cholesterol and triglyceride content, can be seen in Figure 5. In general, an alteration of the lipid profile after FFA exposure was observed, but no variation in free PA or oleic and stearic acid content was detected (fold-change value of 1×, Figure 5). Free LA presence was 24× fold-over after exposure. Therefore, such variation is not as marked as that observed in the total FA analysis (Section 2.5.), which may indicate that, after uptake, the FFA is transformed into another lipid (e.g., triglycerides). Indeed, a four-fold increase in triglycerides (TGs) was observed, suggesting that the FFAs are being esterified into TGs. These results also revealed a two-fold increase in free cholesterol, whose toxicity in hepatocytes can impair inflammation and posterior fibrosis, leading to liver damage [3]. Overall, the exposure to FFA mixture alters the lipid profile of HepG2 cells, and the observed TG increment is consistent with the LA transformation into a more stable form (i.e., TG).

### 2.7. Triglycerides Analysis by High Performance Liquid Chromatography—Evaporative Light Scattering Detector (HPLC-ELSD)

**Figure 6:**
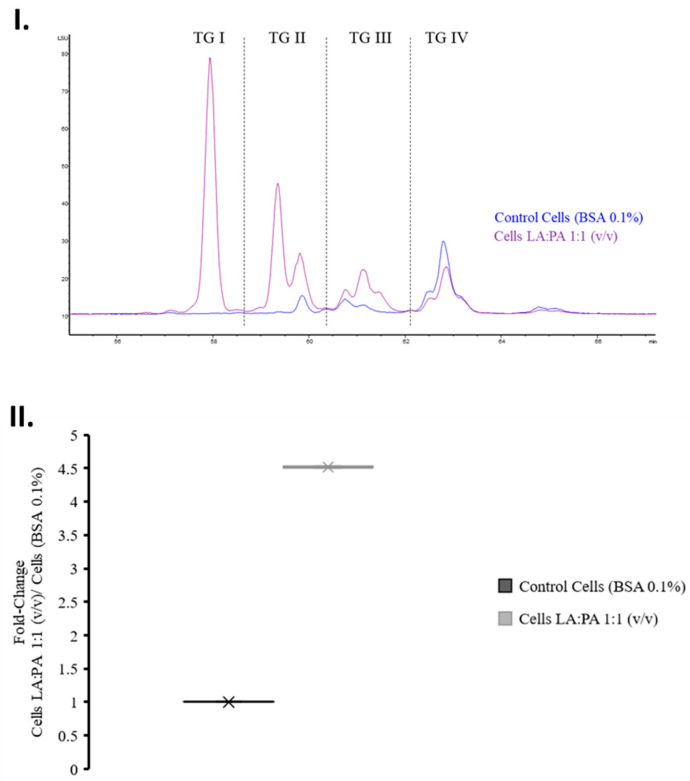
(**I**) Chromatogram showing triglyceride region using HPLC-ELSD analysis after FFA exposure [Blue: control cells vs. purple: LA:PA 1:1 (*v*/*v*)]. (**II**) Fold change variation of the TG content LA: linoleic acid, PA: palmitic acid, TG: triglyceride, BSA: bovine serum albumin.

Different triglyceride groups were detected by HPLC-ELSD analysis (Figure 6I) of the treated cells, and the fold-change values of the triglyceride content determined after FFA exposure [Control Cells vs. LA:PA 1:1 (*v*/*v*)] (Figure 6II) were calculated. The results were in accordance with those obtained by GC-MS, which indicated a four-fold increase in the content of TGs in cells treated with FFA (Figure 5).

### 2.8. Triglyceride Analysis by Liquid Chromatography Coupled with Electrospray Ionization—Quadrupole—Time of Flight (LC-ESI-qTOF) Mass Spectrometry

Previous results revealed that 1 mM LA:PA at 1:1 (*v*/*v*) exposure increased intracellular TG accumulation. The analysis of the TG species was performed by LC-ESI-qTOF and the isotopic pattern of the molecular ion, as well as its MS^2^ fragmentation, which allowed TG identification by comparison with the reference spectra (used database: MS-Finder v3.52 and Lipid Maps^®^).

**Figure 7:**
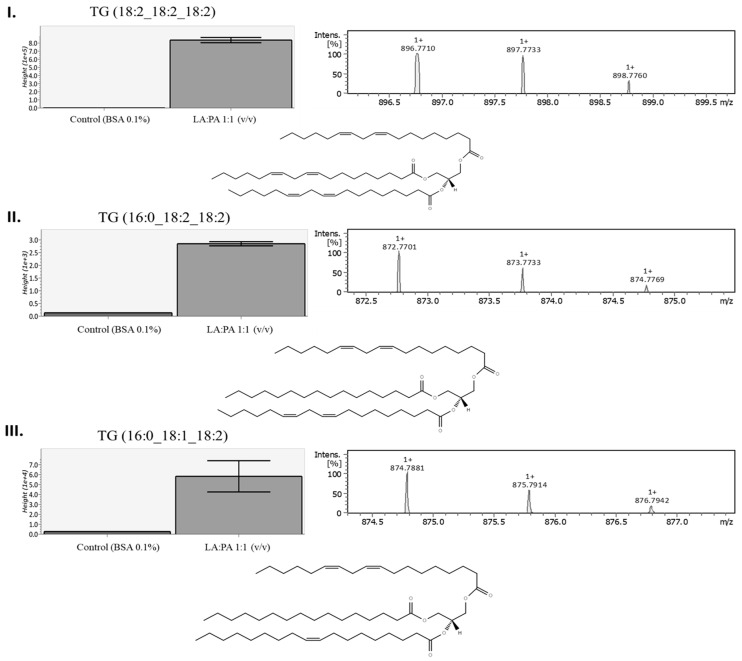
Boxplot, precursor ion isotopic pattern and molecular structure example of possible identified triglycerides by LC-ESI-qTOF after FFA exposure [control cells vs. LA:PA 1:1 (*v*/*v*)]. (**I**) TG C18:2, C18:2, C18:2; (**II**) TG C16:0, C18:2, C18:2 and (**III**) TG C16:0, C18:1, C18:2; FFA: free fatty acids, LA: linoleic acid, PA: palmitic acid. Data treatment was performed using MS-Finder v3.52, MS-Dial v4.90 and LipidMaps^®^.

As can be seen in Figure 7, three species with significant variation (*p* < 0.05) were detected, and data suggested that there was a predominance of LA in the structure of the identified TGs. In general, PA was also present, but only in two of the identified species and in one of the *sn* positions (i.e., TG C16:0, C18:2, C18:2 and TG C16:0, C18:1, C18:2). Thus, results revealed that the presence of TG C18:2, C18:2, C18:2; TG C16:0, C18:2, C18:2 and TG C16:0, C18:1, C18:2 was an effect from the LA and PA exposure and further accumulation.

## 3. Discussion

Fatty acids such as linoleic acid (LA) and palmitic acid (PA) have different physical and biological properties. Thus, LA is an essential fatty acid, and PA is the precursor of long chain fatty acids, such as oleic acid [16,20]. The present work revealed their different accumulation in hepatocytes, being preferential for LA. Such evidence was established by the intracellular detection of LA and long-chain polyunsaturated fatty acids (PUFA), such as arachidonic acid (20:4 n-6). Furthermore, the neutral lipids staining was higher for higher LA concentrations, indicating intracellular accumulation of lipid species. This accumulation also resulted in a four-fold triglyceride (TG) increase, suggesting that the free fatty acids are being esterified into TGs. Besides, previous research works by other authors evidenced impact on cellular energy metabolism caused by lipid accumulation using HepG2 cells under high-glucose requirements [22]. 

Additionally, a two-fold increase in cholesterol was observed. This increase can contribute to cell damage since it is established that free cholesterol is hepatotoxic, inducing inflammation and fibrosis [3,18].

The hepatocyte protection mechanism linked to lipo-toxicity of FFA [23] is generally balanced by FA esterification with glycerol to produce less-toxic TGs [10]. Consequently, the accumulation of intracellular triglyceride and cholesteryl esters is characteristic of hepatic steatosis [16,22]. Other studies showed that, after unsaturated and saturated FA exposure, lipid accumulation in hepatocytes was higher when using unsaturated FA [8,12,16].

Akazawa et al. (2018) described that the predominance of saturated FFAs in hepatocytes can decrease cellular membrane fluidity and induce ER stress, causing cell death [10]. On the other hand, other studies reported that oxidized linoleic acid (unsaturated FFA) metabolites induce liver mitochondrial dysfunction and apoptosis [17]. Nevertheless, current results revealed that LA and PA contribute to cellular apoptosis, but higher percentage of cell death was observed after PA exposure. Moreover, LA and PA induced ROS production, but at different levels, with LA being the fatty acid with major contribution to this effect. Several research works have described that unsaturated FFA can easily trigger ROS production and consequently epoxy- and hydroxy- fatty acid production [4,10,17]. For example, high levels of n-6 PUFA as LA can led towards inflammatory and oxidative stages of NAFLD [18]. Overall, intracellular FA accumulation can result in uncomplete conversion to TGs or β-oxidation, which generate toxic lipids and cell damage [11].

## 4. Materials and Methods

### 4.1. Materials and Chemicals

For culture assays, Dulbecco’s Modified Eagle Medium (DMEM), fetal bovine serum (FBS), and penicillin–streptomycin antibiotic were purchased from Thermofischer, Waltham, MA, USA. HepG2 (HB-8065™) cells were purchased from American Type Culture Collection (ATCC). PrestoBlue (Thermofischer, Waltham, MA, USA). Fatty Acid Free BSA was purchased from Merck (Darmstadt, Germany). Dimethyl sulfoxide was purchased (DMSO) (Molecular Biology Grade, Merck (Darmstadt, Germany)). For total cellular protein quantification, Pierce™ BCA Protein Assay Kit was purchased from Thermofischer (Waltham, MA, USA).

Palmitic Acid/Hexadecanoic Acid (>99%) was purchased from Larodan (Stockholm, Sweden), and linoleic acid (>99%) was purchased from Merck (Darmstadt, Germany).

For GC-MS analysis, all the samples were derivatized with N, O-Bis(trimethylsilyl) trifluoroacetamide with 1% trimethylchlorosilane (BSTFA), purchased from Merck (Darmstadt, Germany). Regarding LC-ESI-qTOF analysis, all reagents were LC-MS grade, and isopropanol (IPA), acetonitrile (ACN), formic acid, and ammonium formate were purchased from VWR (Radnor, PA, USA).

For HPLC-ELSD analysis, chloroform (HPLC grade, ≥99.8%) was purchased from Thermofischer (Waltham, MA, USA), tetrahydrofuran (THF) (HPLC grade, ≥99.9%) was purchased from Merck (Darmstadt, Germany), ultra-pure water was obtained through a Milli-Q system (Merck Millipore, Burlington, MA, USA), and acetic acid (HPLC grade) was obtained from Carlo Erba Reagents (Val de Reuil, France).

The reagents used for lipid extraction were obtained as follows: dichloromethane (DCM) (HPLC grade, ≥99.9%) and n-Hexane (≥97%) from VWR Chemicals (Radnor, PA, USA), Methanol (HPLC grade, ≥99.9%) from Honeywell (Charlotte, NC, USA), sodium methoxide (5.4 M, 30 wt.% solution in Methanol) from Thermofischer (Waltham, MA, USA), sulfuric acid (95.0–97.0%) from Honeywell (Charlotte, NC, USA), and dimethylformamide (DMF) (HPLC grade, ≥99.5%) from Thermofischer (Waltham, MA, USA).

### 4.2. Linoleic Acid (LA) and Palmitic Acid (PA) Preparation

Stock solutions of each fatty acid were prepared at 20 mM in DMEM with 1% of fatty acid free BSA. For PA, the stock solution was heated at 75 °C for 10 min and a further 15 min on a Bandelin Sonorex Super RK 106 Ultrasonic Bath (Berlin, Germany) to improve dissolution. Fatty acids mixture was diluted to 1 mM final concentration using different ratios of LA:PA (1:0, 1:1 and 0:1 *v*/*v*) and then filtered using 0.20 µm-pore size membrane.

### 4.3. Cell Culture

Hepatocellular carcinoma HepG2 cells were kept in culture in Dulbecco’s Modified Eagle Medium (DMEM) supplemented with 10% of FBS and 1% of Penicillin-streptomycin Antibiotic at 37 °C, with 5% of CO_2_ in a humidified atmosphere. HepG2 cells were used at 75% confluency.

#### 4.3.1. Presto-Blue

Metabolic inhibition of lipidic extracts on HepG2 were evaluated using PrestoBlue assay according to manufacturer’s instructions. Cells were seeded at 1 × 10^4^ cells/well in 96-well plates and then exposed to FFA diluted in DMEM with 0.1% fatty acid free BSA for 24 h, in quadruplicates. Wells with media supplemented with LA and PA (without cells) were used to subtract a possible influence of the samples in the PrestoBlue fluorescence signal. Cells treated with 10% DMSO were used as negative control. After incubation, PrestoBlue reagent was added to the media and incubated for 2 h. The fluorescence signal was read in a Synergy H1 microplate reader (BioTek, Instruments, Inc., Winooski, Vermont, USA). Results were expressed in percentage of metabolic inhibition in comparison to cells without treatment. At least two independent experiments were performed.

#### 4.3.2. Flow Cytometric Analysis of LA and PA Effect on Apoptotic Stages

Cells were seeded at 3 × 10^5^ cells/well in 24-well plates and then exposed to FFA for 24 h, in duplicates. After 24 h, cells were washed twice using phosphate-buffered saline (PBS), trypsinized, and washed again with PBS twice by centrifugation (1500 rpm, 5 min, 22 °C). To perform the staining procedure, fluorochrome-labeled Annexin V was used to precisely target apoptotic cells. The apoptotic and necrotic events of cells treated with the different fatty acids (LA and PA) were determined using the Biolegend FITC Annexin V Apoptosis Detection Kit with 7-AAD Cat. 640922 (San Diego, CA, USA), according to the manufacturer’s instructions and analyzed by flow cytometry using a BD Accuri™ C6 Plus Flow Cytometer (New Jersey, USA).

Results are expressed in percentage of live (Av^−^ 7AAD^−^), early apoptotic (Av^+^ 7AAD^−^), necrotic (Av^−^ 7AAD^+^), and dead (Av^+^ 7AAD^+^) cells.

#### 4.3.3. Assessment of Intracellular Reactive Oxygen Species (ROS)

The intracellular generation of ROS in HepG2 cells was determined using the 2′,7′-dichlorofluorescein diacetate (DCFDA) (D6883, Thermofischer) probe. Before the FFA exposure, cells were seeded in black, Thermofisher 96 clear-bottom well plate (Waltham, MA, USA) at 1 × 10^5^ cells/mL and added to each well 100 μL of the appropriate sample (FFA mixture) solutions and incubated during 24 h. Afterwards, the DCFDA probe was prepared and added to the final concentration of 25 μM to each well. The fluorescence of the dye was measured immediately (0 h) using Synergy H1 microplate reader (BioTek, Instruments, Inc.) and for 1, 2, 3, 4 and 5 h of probe incubation at 495/529 nm (Ex./Em.) (Adélia Mendes, personal communication, 2022).

### 4.4. Determination of Total Lipid Accumulation by Oil Red O Staining (ORO)

The neutral lipid content accumulation was evaluated according to the method described by Forbes-Hernández et al. (2017) [24], with some modifications. Cells were seeded at a density of 3 × 10^5^ cells/mL (1 mL) in 24-well plates for 24 h and then treated with the FFAs mixtures (500 µL of mixture per well). After 24 h of exposition, cells were washed twice using PBS (500 µL per well) and fixed in 4% Paraformaldehyde (PFA) diluted in PBS for 30 min to 1 h.

Oil Red O (ORO) stock solution was prepared in 2-propanol at 3.5 mg/mL and filtered through a 0.45 μm pore size hydrophobic membrane. Then, a working solution was set up using three parts of the ORO stock solution and two parts of water. Fatty acid treated and control cells were stained with Oil Red O working solution for 15 min at room temperature (250 µL per well), and PBS was later used to remove the non-stained ORO by washing wells three consecutively times.

Afterwards, cell images were taken using ZEISS (Jena, Germany) Optical Microscope Axiocam 208 Color Camera. For semi-quantitative analysis of the stained lipids, 250 µL per well of 2-propanol was added to the 24-well plate containing cells, and then the solution was transferred to a 96-well microplate, and the absorbance was measured at 510 nm in Synergy H1 microplate reader (BioTek, Instruments, Inc., Winooski, Vermont, USA). The absorbance was normalized by cellular protein quantification at 562 nm using Pierce™ BCA Protein Assay Kit.

### 4.5. Lipid Extraction of the Cellular Content

After 24 h of fatty-acid exposure, cells were observed, and then the supplemented fatty acid medium was aspired, and cells were once washed with PBS. Cells were trypsinized using TrypLE for 10 min and transferred to a falcon tube to be centrifuged (1500 rpm, 5 min, 22 °C). The obtained pellet was collected, and PBS was added and centrifuged (1500 rpm, 5 min, 22 °C), and the supernatant discarded afterwards.

The total lipid content of each pellet was acquired using methyl-tert-butyl ether (MTBE) and methanol according to Matyash et al. (2008) extraction procedure [25].

### 4.6. LC-ESI-qTOF

Matyash cell extracts were prepared at a stock solution of 1 mg/mL and dissolved in IPA:ACN [9:1] at 0.20 mg/mL to be further analysed. Additionally, for quality requirements after data acquisition, a quality control (QC) sample was prepared using a pool of the samples and injected in positive mode on an UHPLC instrument (Elute; Bruker, Billerica, MA, USA) equipped with an Acquity UPLC BEH C18 (17 µm) pre-column (Waters, Milford, MA, USA), an Intensity Solo 2 C18 (100 × 2.1 mm) column (Bruker, Billerica, MA, USA), and coupled with an UHR–QTOF detector (Impact II; Bruker). Injection method was based on conditions reported by Sarafian et al. (2014) [26] and Calderón et al. (2019) [27], with some modifications. The mobile phases composition was the following: ACN:upH_2_O [6:4, *v*/*v*] (Phase A) and IPA:ACN [9:1, *v*/*v*] (Phase B), both containing 0.1% (*v*/*v*) of formic acid and 10 mM ammonium formate modifiers. Gradient of B phase flow was as follows: 0.0 min—40%; 2.0 min—43%; 2.1 min—50%; 12.0 min—54%; 12.1 min—70%; 18.0 min—99%; 20.0 min—99%; 20.1 min—40%; 22 min—40%. The flow rate was set at 0.4 mL/min, and column temperature was set at 55 °C. Injection volume was 3 µL in positive and 5 µL in negative ionization mode. For MS analysis, the following parameters were applied: end plate offset voltage 500 V, capillary voltage 4500 V (positive ionization) or 3000 V (negative ionization), nebulizing gas pressure of 35 psi, drying gas flow 8 L/min, drying gas temperature 325 °C, quadrupole ion energy 3 eV (positive ionization) or 5 eV (negative ionization), collision energy 10 eV (positive ionization) or 5 eV (negative ionization). Acquisition was performed in an Auto MS/MS scan mode over a mass range of m/z 50–1500. For both ionization modes, an external mass calibration was performed with a solution of IPA:upH_2_O [1:1, *v*/*v*] added with 0.2% (*v*/*v*) formic acid and 0.6% (*v*/*v*) NaOH 1 M, continuously injected at 180 µL/h. The acquired data were treated using MS-DIAL (v 4.90) and compound identification by MS-FINDER (v 3.52).

### 4.7. GC-MS

The Matyash extracts were analysed after derivatization into their trimethylsilyl derivatives. For that, to 1 mg of sample 70 µL of DCM and 60 µL of BSTFA were added. After 30 min incubation at 70 °C, DCM was added to a final volume of 400 µL. Afterwards, the samples were analysed on a GC-MS (triple quadrupole) model EVOQ (Bruker, Karlsruhe, Germany) mass spectrometer, coupled with a Rxi-5Sil MS column (30 m × 250 µm × 0.25 µm) at constant flow of 1 mL/min. Helium was used as carrier gas, as described by Teixeira et al., (2022) [28]. In summary, the injector was set at 330 °C, and the oven temperature at 60 °C. After a 5 min hold, the temperature was increased at 3 °C/min until 330 °C and maintained for more 20 min. The mass spectrometer detector was operated in electron ionization mode (EI) at −70 eV, the source temperature of 280 °C, the transfer line at 300 °C, and a quadrupole in a scan range of 33 to 1000 amu per second. The compound identification was based on the comparison of the obtained mass spectra with the information on the NIST Library (v. 2.3), as well as by comparison with reference compounds.

### 4.8. GC-FID

To determine the fatty acids profile, the performed method was based on Fontes at al. [29]. The obtained washed cell pellets were prepared at 1 mg/mL final concentration in hexane. Glyceryl tritridecanoate (≥99%, Merck) was used as internal standard, followed by 2.26 mL of methanol, and 240 µL of sodium methoxide (5 M). The samples were vortexed and incubated at 80 °C for 10 min. After cooling in ice, 1.25 mL of DMF were added prior to 1.25 mL of sulphuric acid (3 M). Samples were vortexed and incubated at 60 °C for 30 min. Finally, after cooling, samples were vortexed and centrifuged (1250× *g*; 18 °C; 5 min). The organic phase containing fatty acid methyl esters (FAME) was collected to a tared vial and then the hexane (approx. 1 mL) was evaporated and re-suspended in 300 uL of hexane for further analysis in a GC-FID apparatus equipped with a BPX70 column (60 m x 0.25 mm x 0.25 µm, SGE Trajan). The analysis conditions were set as follows: injector temperature 250 °C, split 25:1, injection volume 1 μL; detector (FID) temperature 275 °C; hydrogen was carrier gas at 20.5 psi; oven temperature program: started at 60 °C (held 5 min), then raised at 15 °C/min to 165 °C (held 1 min) and finally at 2 °C/min to 225 °C (held 2 min). The identification of fatty acids was carried out by comparison with a Supelco 37 FAME mixture sample (CRM47885).

### 4.9. HPLC-ELSD

For the triglyceride analysis, the Plante et al. (2011) [30] methodology was used with some modifications. Approximately 3 mg/mL of the cellular Matyash extract were dissolved in methanol: chloroform mixture (1:1, *v*/*v*) was injected in a high-performance liquid chromatograph (HPLC) (1260 Infinity II; Agilent, Santa Clara, CA, USA) equipped with an evaporative light scattering detector (ELSD) and a Zorbax Eclipse Plus C8 column (2.1 × 100 mm; Agilent). The detector operation conditions were set with the evaporator at 30 °C, the nebulizer at 40 °C, and nitrogen as carrier gas with a flow rate set at 1.5 SLM. The mobile phases were prepared and filtered by a 0.20 µm-pore size hydrophobic membrane: A (methanol, water and acetic acid, 750:250:4 (*v*/*v*)) and B (acetonitrile, methanol, tetrahydrofuran, and acetic acid, 500:375:125:4 (*v*/*v*)) in a gradient mode were set between 0–45 min (100% Phase A, 46–59 min—30% Phase A and 70% Phase B, 60–65 min—10% Phase A and 90% Phase B, and 65.10–72 min—100% Phase A). The determination was carried out by injecting 10 µL of sample with a flow rate of 0.5 mL/min with the oven set at 40 °C.

## 5. Conclusions

The reported results showed that PA led to lower metabolic inhibition and, interestingly, when compared to LA, PA was poorly accumulated. Thus, LA was associated with higher accumulation in both FFA and TG forms. Triglyceride variation was analyzed and potential species identified, such as TG C18:2, C18:2, C18:2; TG C16:0, C18:2, C18:2 and TG C16:0, C18:1, C18:2, probably as a protective mechanism to limit FFA impact on cell damage.

In Western diets, LA and PA sources are highly abundant [17,18,19]. The present work showed the importance to balance both PA and LA fatty acids concentrations in HepG2 cells to maintain normal levels of FFAs, cholesterol and TGs. Consequently, therapies that induce fatty acids lipolysis, when it is not possible to control their intake, are currently being studied to induce liver homeostasis and avoid irreversible stages of fibrosis [23]. Although, in this situation, the use of drugs capable of increasing β-oxidation of fatty acids would be useful in controlling lipid accumulation, it still represents a pharmacological intervention that does not solve an underlying problem resulting from an unhealthy lifestyle. Consequently, these treatments can be very helpful in extreme situations (i.e., obesity), but it should not be forgotten that maintaining a healthy diet (i.e., inclusion of vegetables and fruits followed by moderate consumption of animal fats and seed oils), when combined with frequent physical exercise and consistent sleeping routine, would help to minimize some of the observed in vitro effects of fatty acids in this research work.

Future work should study the binding and transmembrane transport of these FFA in HepG2 cells. The identification of other lipid species (e.g., ceramides and oxidized lipids) involved in steatotic hepatocytes may also help to better understand and describe other underlying processes, as well as the study of the inflammatory and ER stress derived from the intracellular presence of LA and PA.

## Figures and Tables

**Table 1:** Fatty acids content (mg FA/g cell pellet) of control cells vs. 1 mM LA:PA 1:1 (*v*/*v*), obtained by GC-FID.

	Control (BSA 0.1%)			LA:PA 1:1 (*v*/*v*)			*p*
			mg FA/g Cell Pellet				
**Myristic Acid** (C14)	3.87	±	0.01	5.61	±	0.01	**
**Palmitic Acid** (C16)	34.81	±	0.03	60.77	±	0.12	**
**Palmitoleic Acid** (C16:1c9)	10.85	±	0.01	7.56	±	0.02	**
**Stearic Acid** (C18)	9.55	±	0.01	17.90	±	0.03	**
**Oleic Acid** (C18:1c9)	26.41	±	0.01	22.62	±	0.05	**
***cis*-11-Vaccenic Acid** (C18:1c11)	15.67	±	0.03	12.15	±	0.02	**
**Linoleic Acid** (C18:2c9c12)	1.12	±	0.01	116.75	±	0.26	**
**γ-Linolenic Acid** (C18:3c6c9c12)		nd		1.07	±	0.02	*
***cis*-11-Eicosenoic Acid** (C20:1c11)	1.12	±	0.01	0.97	±	0.01	*
***cis*-11,14-Eicosadienoic Acid** (C20:2c11c14)		nd		3.78	±	0.03	**
***cis*-8,11,14-Eicosatrienoic Acid** (C20:3c8c11c14)	0.60	±	0.05	2.91	±	0.04	**
**Arachidonic Acid** (C20:4c5c8c11c14)	2.77	±	0.01	4.57	±	0.03	**
**Total Fatty Acids**	106.78	±	0.11	256.66	±	0.54	**

Data represented as mean ± st. dev. (n = 2); * *p* < 0.05; ** *p* < 0.01; nd: not detected; LA: linoleic acid, PA: palmitic acid.

## Data Availability

Not applicable.

## Abbreviations

Acetonitrile (ACN), 2′,7′-dichlorofluorescein diacetate (DCFDA), Dichloromethane (DCM), Dimethyl sulfoxide (DMSO), Dimethylformamide (DMF), Dulbecco’s Modified Eagle Medium (DMEM), Electrospray Ionization-quadrupole-Time Of Flight (ESI-qTOF), Endoplasmic Reticulum (ER), Fetal Bovine Serum (FBS), Fatty Acid (FA), Fatty Acids Methyl Esters (FAME), Free Fatty Acids (FFA), Gas-Chromatography (GC), High Performance Liquid Chromatography—Evaporative Light Scattering Detector (HPLC-ELSD), Isopropanol (IPA), Linoleic Acid (LA), Lipid Droplets (LD), Methyl-tert-Butyl Ether (MTBE), N, O-Bis(trimethylsilyl) Trifluoroacetamide with trimethylchlorosilane (BSTFA), Non-Alcoholic Fatty Liver Disease (NAFLD), Non-Alcoholic Steatohepatitis (NASH), Oil Red O (ORO), Palmitic Acid (PA), Peroxisome Proliferator-Activated Receptor (PPAR), Phosphate Buffered Saline (PBS), Polyunsaturated Fatty Acids (PUFA), Relative Fluorescence Units (RFU), Reactive Oxygen Species (ROS), Tetrahydrofuran (THF), Triglycerides (TG).
